# Global trends in perioperative stroke research from 2003 to 2022: a web of science-based bibliometric and visual analysis

**DOI:** 10.3389/fneur.2023.1185326

**Published:** 2023-05-31

**Authors:** Shunpan Ji, Yue Shi, Xiaojing Fan, Tian Jiang, Xiaoming Yang, Tianzhu Tao, Bo Ye

**Affiliations:** ^1^Department of Anesthesiology, Air Force Medical Center, Beijing, China; ^2^Graduate School of China Medical University, Shenyang, China

**Keywords:** anesthesia, bibliometric, perioperative period, stroke, surgery, visualized study

## Abstract

**Background:**

Perioperative stroke is a potentially devastating complication in surgical patients, which has attracted global attention. This retrospective bibliometric and visual analysis evaluates the status and global trends in perioperative stroke research.

**Methods:**

Papers published between 2003 and 2022 were retrieved from the Web of Science core collection. Extracted data were summarized and analyzed using Microsoft Excel and further bibliometric and co-occurrence analyses were conducted using VOSviewer and CiteSpace software.

**Results:**

Publications on perioperative stroke have increased over the years. The USA topped the list of countries with the highest number of publications and citations, while Canada had the highest mean citation frequency. The Journal of Vascular Surgery and Annals of Thoracic Surgery had the highest number of publications and citation frequency for perioperative stroke. Regarding authors, Malas, Mahmoud B. contributed the most publications to the field, and Harvard University had the highest number of publications (409 papers). Based on an overlay visualization map, timeline view, and the strongest strength burst of keywords, “antiplatelet therapy,” “antithrombotic therapy,” “carotid revascularization,” “bleeding complications,” “postoperative cognitive dysfunction,” “intraoperative hypotension,” “thrombectomy,” “cerebral revascularization,” “valve surgery,” “tranexamic acid,” and “frozen elephant trunk” were trending topics in perioperative stroke research.

**Conclusion:**

Publications regarding perioperative stroke have experienced rapid growth in the past 20 years and are likely to continuously increase. Research on perioperative antiplatelet and antithrombotic, cardiovascular surgery, postoperative cognitive dysfunction, thrombectomy, tranexamic acid, and frozen elephant trunk has attracted increasing attention, and these topics are emerging hotspots of present research and possible candidates for future research.

## 1. Introduction

Stroke is characterized by brain cell death due to ischemia with indications of irreversible damage. Its diagnosis is established by neuropathology, neuroimaging, or clinical evidence (1). Although such lesions may result in critical functional impairments, a small infarct may fail to manifest clinically (i.e., covert stroke). Perioperative stroke is defined as the onset of cerebral infarction within 30 days after surgery (2). The prevalence of overt perioperative stroke ranges from approximately 0.1 to 2% and is determined by various risk factors (3). A covert stroke occurs in approximately 7% of non-cardiac surgical patients, aged >65 years (4). An epidemiological database demonstrated that the prevalence of perioperative stroke is increasing (5). In addition, a high risk of long-term cognitive impairment, visible in patients with covert stroke, has been observed in patients with a clinical diagnosis of stroke who presented with symptoms such as delirium and postoperative cognitive dysfunction (4, 6). Owing to delayed recognition of perioperative stroke which results in a lack of appropriate and timely intervention, non-cardiac surgical patients with postoperative stroke have >80% risk of death or severe disability upon their discharge from prolonged medical care (7). Hence, perioperative stroke is a serious medical condition that should be actively addressed by public health practitioners.

Publications are essential for research trend examination. Consequently, bibliometrics provides a valuable statistical and analytical tool that can be used to perform a qualitative and quantitative evaluation of research trends based on the bibliometric characteristics of publications databases (8). This approach analyzes the evolution of a specific field and appraises relevant contributions made to it by countries, institutions, authors, and journals. CiteSpace and VOSviewer are recently popular Java applications for visualizing and analyzing trends and patterns in scientific literature. These tools provide various functions to facilitate the understanding and interpretation of network patterns and historical patterns, including identifying the fast-growth topical areas, finding citation hotspots in the land of publications, decomposing a network into clusters, geospatial patterns of collaboration, and unique areas of international collaboration.

This study aimed to conduct a comprehensive and systematic literature-based metric data analysis of perioperative stroke-related studies. To clarify the direction of perioperative stroke research and provide a basis for the prevention and treatment of related complications, this study summarized and highlighted the state of worldwide research on the condition over the past 20 years.

## 2. Methods

### 2.1. Data sources and search strategy

**Figure 1:**
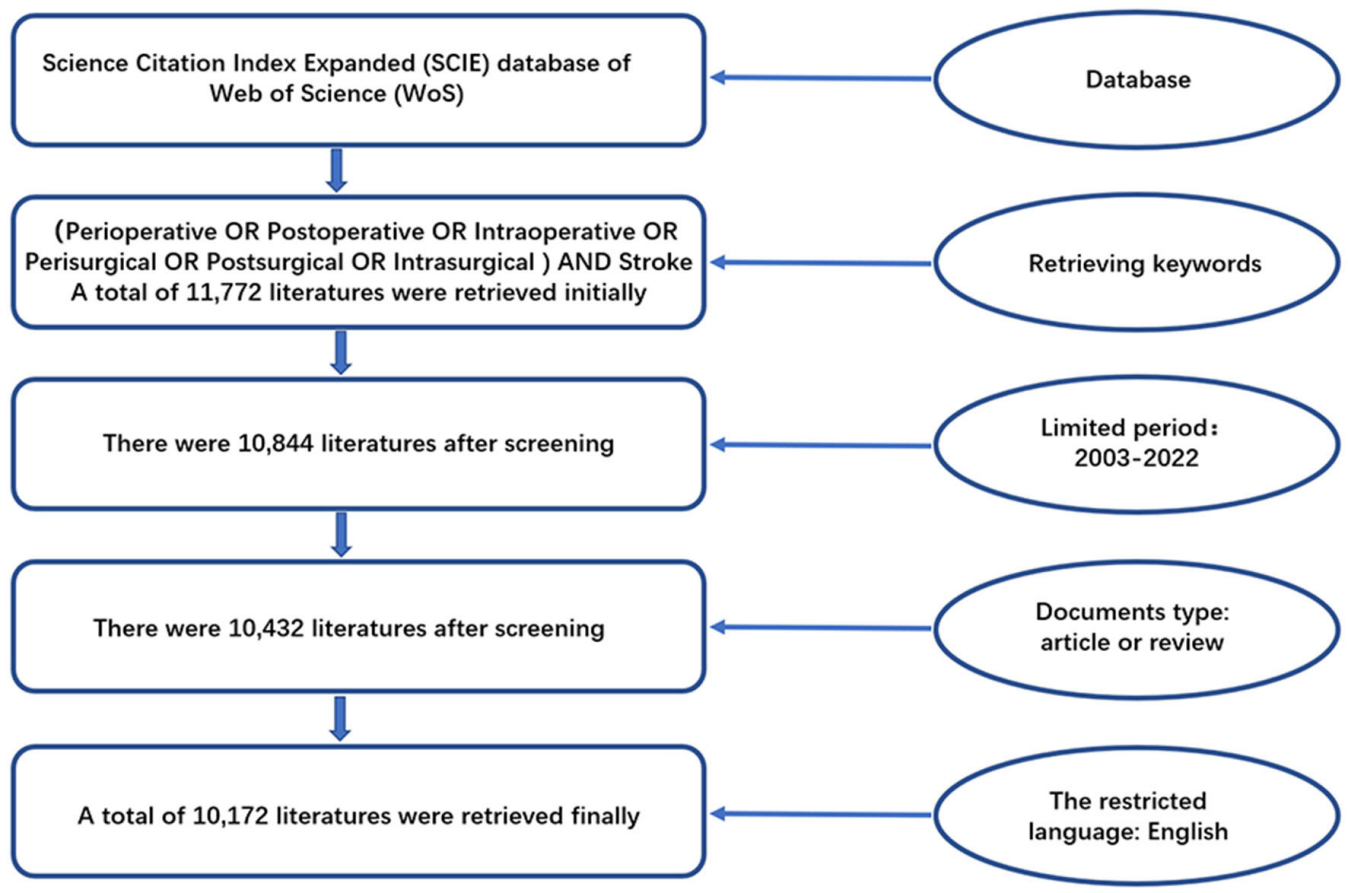
Flow chart of literature search, filtering, and selection of included publications.

The initial search was performed on 10 January 2023 and updated on 4 February 2023, from the Science Citation Index Expanded database of Web of Science (WoS) by entering the following keywords: (perioperative OR postoperative OR intraoperative OR perisurgical OR postsurgical OR intrasurgical) AND “stroke”—in the topic field. A total of 11,772 publications were initially retrieved. After restricting the timespan to 2003–2022, 10,844 publications remained. Only reviews and articles were included, leaving 10,432 publications after the screening. Finally, the English language restriction was applied for a final total of 10,172 publications (Figure 1).

### 2.2. Data extraction and visualization

The retrieved publications were compiled and exported into “plain text” files. The exported records comprised “full records and references cited” and the extracted data included authors, publication year, journals, H-index, institutions, and countries. The data were analyzed using Microsoft Excel 2019 (Microsoft Corporation, Santa Rosa, CA, USA), VOSviewer (version 1.6.18; Leiden University), and CiteSpace (6.1.R6).

Microsoft Excel 2019 was used to assemble and sequence every publication feature. As popular knowledge mapping tools, the VOSviewer and CiteSpace—operated using a Java program—provided strong data visualization capabilities. Every record in the WoS database was imported into VOSviewer by using co-authorship, co-citation, and co-occurrence analysis. Burst detection and timeline view were performed on the keywords and the strongest keywords were extracted by CiteSpace.

Co-authorship analysis was used to connect two elements who co-authored an article to reveal a specific condition of the collaboration network (9). Co-citation analysis was used to examine the relationship between the two documents by determining the frequency of simultaneous citations of other documents (10). Co-occurrence analysis was used to calculate every keyword to identify high-frequency terms and research directions. Visualization of links across countries, institutions, and authors was accomplished by using weighted total link strength (TLS) lines. TLS signified the strength of linkages between objects; the higher the TLS, the greater the weight given to the linkage drawing in the visual analysis.

## 3. Results

### 3.1. Number of publications and their trend

**Figure 2:**
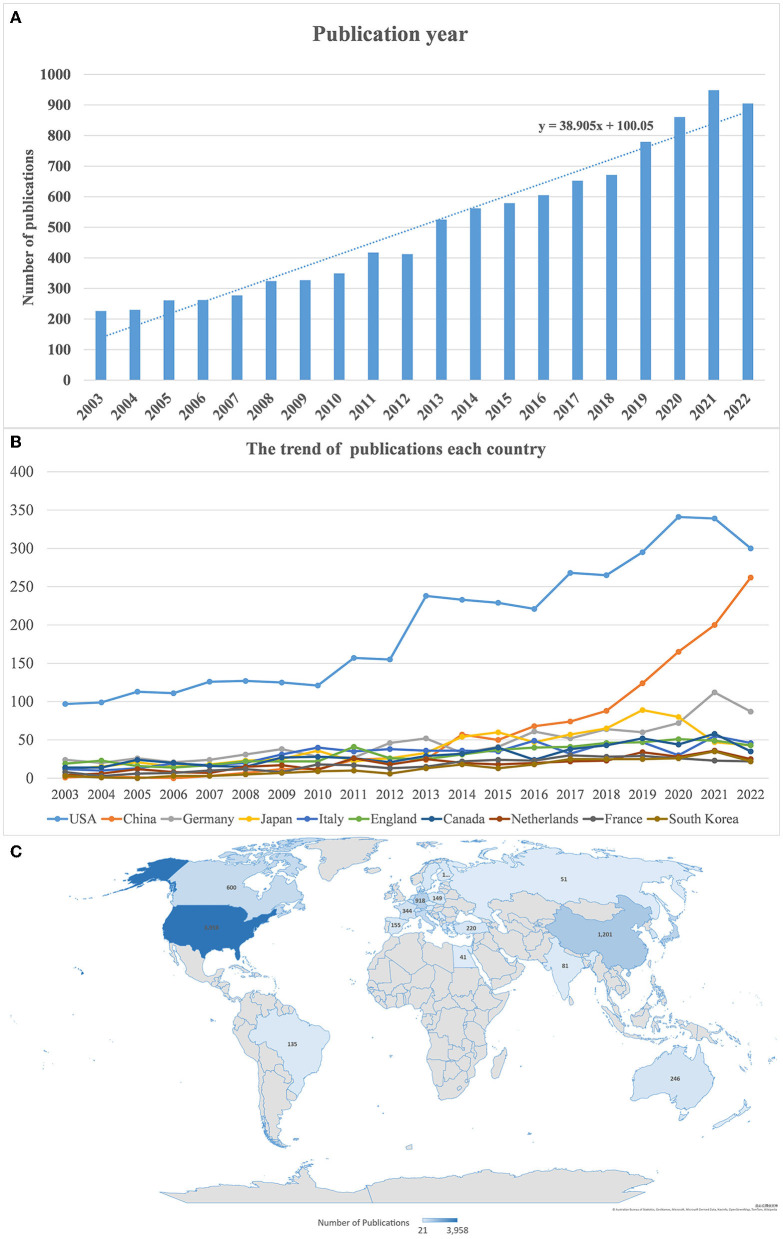
**(A)** Annual number of publications. **(B)** Annual publication trend in every country. **(C)** Distribution of perioperative stroke research by country from 2003 to 2022.

The number of publications on perioperative stroke increased annually from 2003 to 2022. They peaked in 2021 with 948 (9.32%) studies (Figure 2A). Despite slight fluctuations over the past two decades, the general publication trend has been on the rise. Based on the growth model equation in Microsoft Excel (Y = 38.905X + 100.05), approximately 1,267 papers are projected to be published by 2032. Overall, these data indicate that research on perioperative stroke has been a focus area and the value of mining is increasing.

### 3.2. Country analysis

Over the past two decades, 42 countries have published over 20 articles on perioperative stroke. Figure 2C presents the geographical distribution of these publications. The prolific countries include North America, Europe, and some Asian countries, mainly concentrated in the Northern Hemisphere. Notably, the USA was responsible for the highest number of publications (*n* = 3,958), followed by China (*n* = 1,201) and Germany (*n* = 918). A list of 10 countries credited with the highest number of publications on perioperative stroke is presented in Table 1. The USA had the highest H-index (132), followed by Canada (84), England (78), and Germany (77). In addition, it had the highest number of citations (*n* = 114,598), followed by Canada (*n* = 31,525), Germany (*n* = 28,143), and China (*n* = 12,939). Publications from Canada had the highest mean number of citations (*n* = 52.54), followed by those from England (*n* = 46.40), the Netherlands (*n* = 34.09), and Germany (*n* = 30.66).

**Table 1:** Ten most prolific countries.

**Country**	**Number (%)**	**H-index**	**Total times cited**	**Average per term**
USA	3,958 (38.9%)	132	114,598	28.95
China	1,201 (11.8%)	48	12,939	10.77
Germany	918 (9.0%)	77	28,143	30.66
Japan	788 (7.7%)	45	11,228	14.25
Italy	647 (6.4%)	62	15,834	24.47
England	632 (6.2%)	78	29,327	46.40
Canada	600 (5.9%)	84	31,525	52.54
Netherlands	375 (3.7%)	51	12,782	34.09
France	344 (3.4%)	46	7,974	23.18
South Korea	267 (2.6%)	27	3,118	11.68

**Figure 3:**
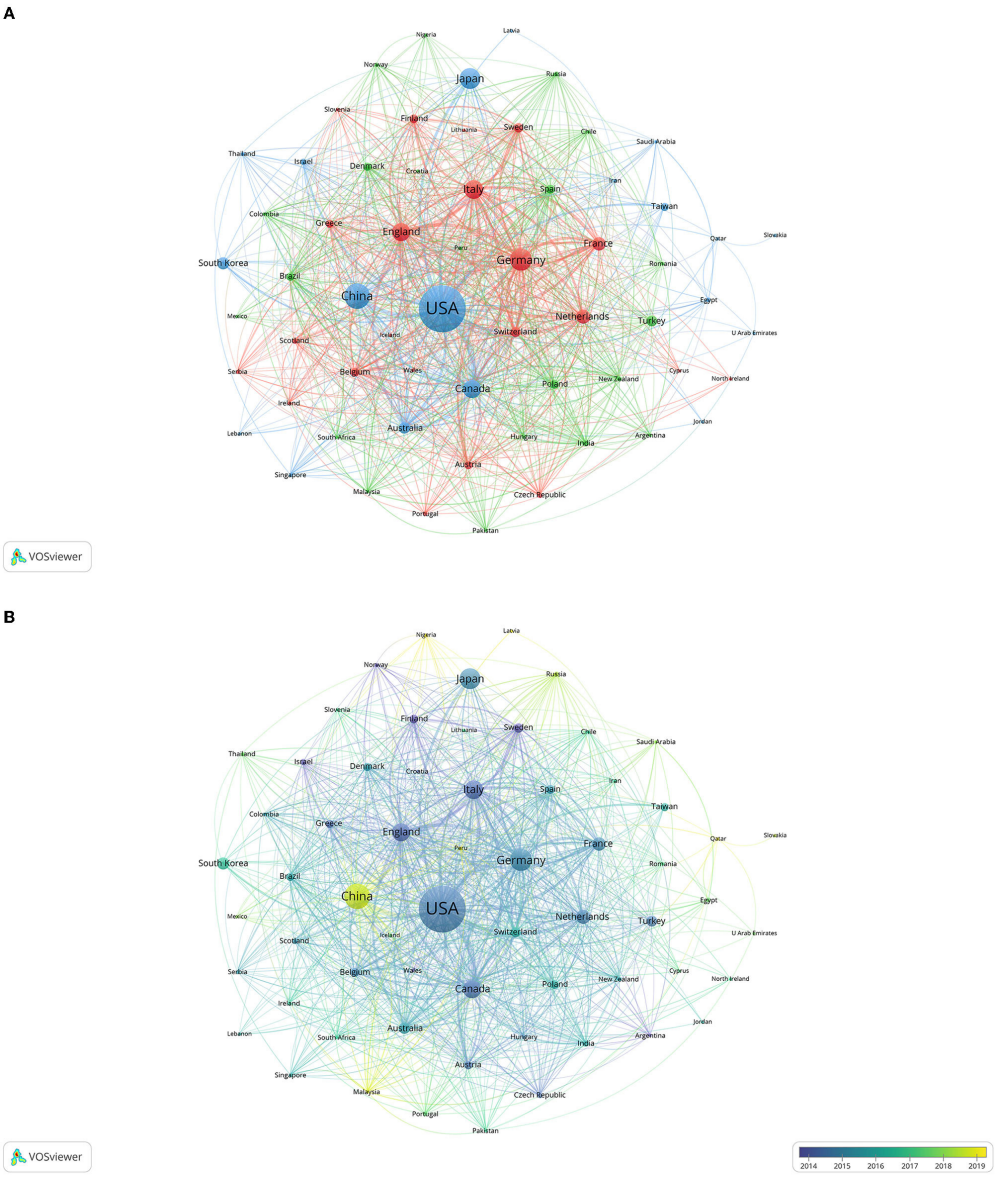
Co-authorship analysis of countries. Link thickness indicates collaboration strength and node size denotes the number of publications by every country. **(A)** Network visualization; **(B)** Overlay visualization.

For an in-depth overview of the collaboration between countries, we used VOSviewer software to visualize the co-authorship analysis. A total of 63 countries had at least five publications. Each node in the VOSviewer represented a country, and the node radius increased with the number of publications. The connections between nodes represented the collaborative relationships between individual countries, and the link thickness was positively associated with the collaboration strength. The five countries with the highest TLS were the USA (1,465), Germany (877), England (846), Italy (769), and Canada (736) (Figures 3A, B). Remarkably, the USA had the highest interpersonal collaboration with different countries as evidenced by its location at the center of the network.

### 3.3. Institution analysis

Between 2003 and 2022, 40 institutions published over 100 articles on perioperative stroke. The 10 centers with the highest number of publications are presented in Table 2. They included eight research institutions from the USA and one each from Canada and China. Harvard University had the highest number of publications (*n* = 409), followed by the University of California System (*n* = 295) and Mayo Clinic (*n* = 284). The three institutions also had the highest H-indices—60 (Harvard University), 49 (the University of California System), and 49 (Mayo Clinic).

**Table 2:** Ten most prolific institutions.

**Institutions**	**Record count (%)**	**Country**	**H-index**	**Sum of times cited**	**Average per item**
Harvard University	409 (4.0%)	USA	60	16,483	40.3
University of California System	295 (2.9%)	USA	49	8,579	29.08
Mayo Clinic	284 (2.8%)	USA	49	9,480	33.38
Harvard Medical School	244 (2.4%)	USA	42	9,321	38.2
Cleveland Clinic Foundation	225 (2.2%)	USA	48	9,439	41.95
University of Toronto	216 (2.1%)	Canada	49	9,563	44.27
Johns Hopkins University	206 (2.0%)	USA	39	8,272	40.16
Pennsylvania Commonwealth System of Higher Education Pcshe	206 (2.0%)	USA	35	7,167	34.79
Capital Medical University	196 (1.9%)	China	20	1,475	7.53
University of Pennsylvania	188 (1.8%)	USA	49	8,459	44.99

**Figure 4:**
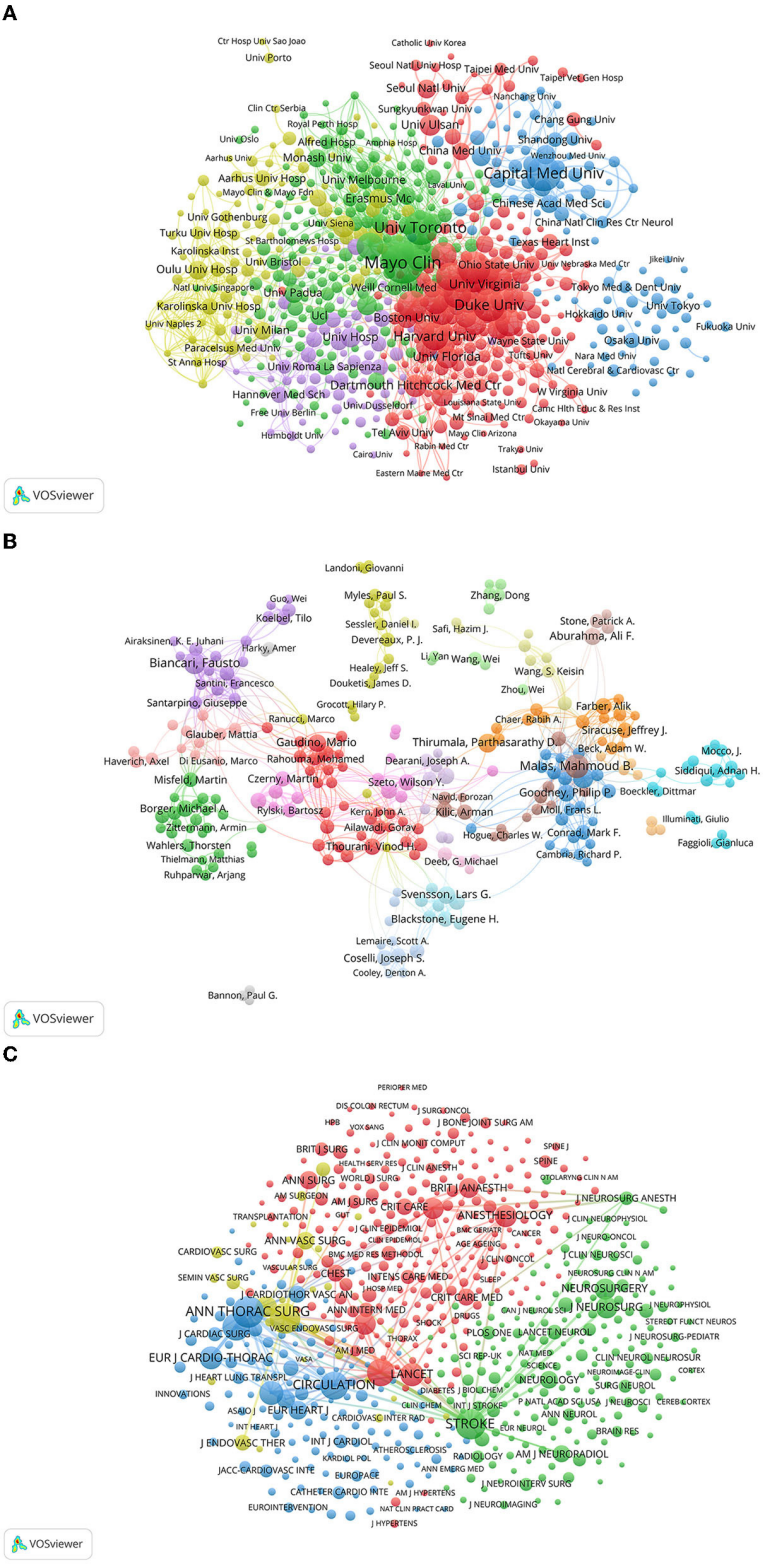
**(A)** Co-authorship analysis of institutions. The nodes of the circle represent the institutions and the links denote collaborations between them. The node size relates to the number of published articles and collaboration strength is indicated by link thickness. **(B)** Co-authorship analysis of authors. **(C)** Co-citation analysis of journals. The links between nodes indicate the citation frequency of the articles. Journals with higher co-citation frequency have larger nodes.

We selected 923 institutions for a visualization based on a minimum of five publications and constructed a co-authorship network based on the number of publications and relationships at each institution. The five centers with the highest TLS were the University of Toronto (606), McMaster University (463), Cleveland Clinic (396), Harvard Medical School (377), and Duke University (365) (Figure 4A). The connection density in the collaboration network diagram demonstrated that institutions from North America and Europe often collaborated closely.

### 3.4. Author analysis

A list of the 10 most prolific authors is presented in Table 3. These authors published 443 publications, accounting for 4.35% of the total. Malas, Mahmoud B. from the University of California, San Diego (USA) contributed the highest number of publications (*n* = 72), followed by Biancari, Fausto (*n* = 68), Schermerhorn, Marc L. (*n* = 51), De Borst, Gert J (*n* = 43), and Gaudino, Mario F.L. (*n* = 39). Among the 10 authors, Joseph E. Bavaria had the highest H-index (24) and a mean number of citations (*n* = 56.49). Furthermore, most of the top 10 authors were from the USA. This suggests that there are more researchers in the USA who focus on perioperative stroke.

**Table 3:** Ten most prolific authors.

**Author**	**Country**	**Number of publications**	**H-index**	**Average citation**	**Total times cited**	**Institution**
Malas, Mahmoud B.	USA	72	18	13.49	971	University of California San Diego
Biancari, Fausto	Italy	68	23	24.99	1,699	Helsinki University Hospital
Schermerhorn, Marc L.	USA	51	17	18.06	921	Beth Israel Deaconess Medical Center
De Borst, Gert J.	Netherlands	43	17	20.07	863	Utrecht University Medical Center
Gaudino, Mario F. L.	USA	39	15	13.46	525	Cornell University
Goodney, Philip P.	USA	39	19	20.92	816	White River Junct VA Med Ctr
Joseph E. Bavaria	USA	37	24	56.49	2,090	University of Pennsylvania
Pochettino, Alberto	USA	34	22	46.29	1,574	Mayo Clinic
Borger, Michael A.	USA	30	21	71.1	2,133	Heart Center Leipzig GMBH
Farber, Alik	USA	30	10	8.63	259	Boston University

A co-authorship map was drawn to indicate the authors who had collaborated on perioperative stroke research. The size of the circle refers to the number of articles published by the author. The connection between the nodes reveals a collaborative relationship between them. A total of 326 authors had published at least 10 articles. The five authors with the highest TLS were Malas, Mahmoud B. (156), Gaudino, Mario F.L. (135), Biancari, Fausto (134), Santarpino, Giuseppe (127), and Mariscalco, Giovanni (116) (Figure 4B). There are multiple potential cooperation teams in the author's cooperation network. Nevertheless, the collaborative network of authors is scattered, and cross-border cooperation needs to be strengthened.

### 3.5. Journal analysis

A list of the 10 most prolific journals with the highest number of publications on perioperative stroke is presented in Table 4. The Journal of Vascular Surgery had the highest number of articles on the condition (*n* = 626), followed by the Annals of Thoracic Surgery (*n* = 517), the Journal of Thoracic and Cardiovascular Surgery (*n* = 336), and the Annals of Vascular Surgery (*n* = 320). The Journal of Vascular Surgery had the highest number of citations (*n* = 17,872), followed by the Annals of Thoracic Surgery (*n* = 17,529), the Journal of Thoracic and Cardiovascular Surgery (*n* = 14,437), and the European Journal of Cardio-thoracic Surgery (*n* = 7,654). According to the 2021 Journal Citation Reports, Stroke had the highest impact factor (10.17), followed by the Annals of Thoracic Surgery (5.11) and the Journal of Vascular Surgery (4.86) (Table 4).

**Table 4:** Ten most prolific journals.

**Journal**	**Total publications**	**Times Cited**	**H-Index**	**Average per item**	**IF (2021)**
Journal of vascular surgery	626	17,872	60	28.55	4.86
Annals of thoracic surgery	517	17,529	65	33.91	5.11
Journal of thoracic and cardiovascular surgery	336	14,437	64	42.97	4.53
Annals of vascular surgery	320	3,144	27	9.83	1.60
European journal of cardio-thoracic surgery	293	7,654	47	26.12	4.53
World neurosurgery	234	2,036	23	8.7	2.21
Journal of cardiac surgery	186	1,634	21	8.78	1.78
Journal of cardiothoracic and vascular anesthesia	179	2,478	28	13.84	2.89
Interactive cardiovascular and thoracic surgery	149	1,663	23	11.16	1.98
Stroke	142	7,780	49	54.79	10.17

The articles cited by every publication were represented as nodes in a co-citation visualization network. Co-cited journals were cited by other researchers. Clusters symbolized by different colors were generated based on citation links. The size of the circle indicates the co-citation frequency. The line between the circles represents the co-citation relationship. Thickness and the number of connections with the nodes indicate the link strength between the journals. A total of 623 journals were cited at least 50 times. The five journals with the highest TLS were Stroke (546,381), New England Journal of Medicine (468,018), Annals of Thoracic Surgery (456,554), Circulation (439,048), and Journal of Vascular Surgery (413,991) (Figure 4C).

### 3.6. Keyword analysis

**Figure 5:**
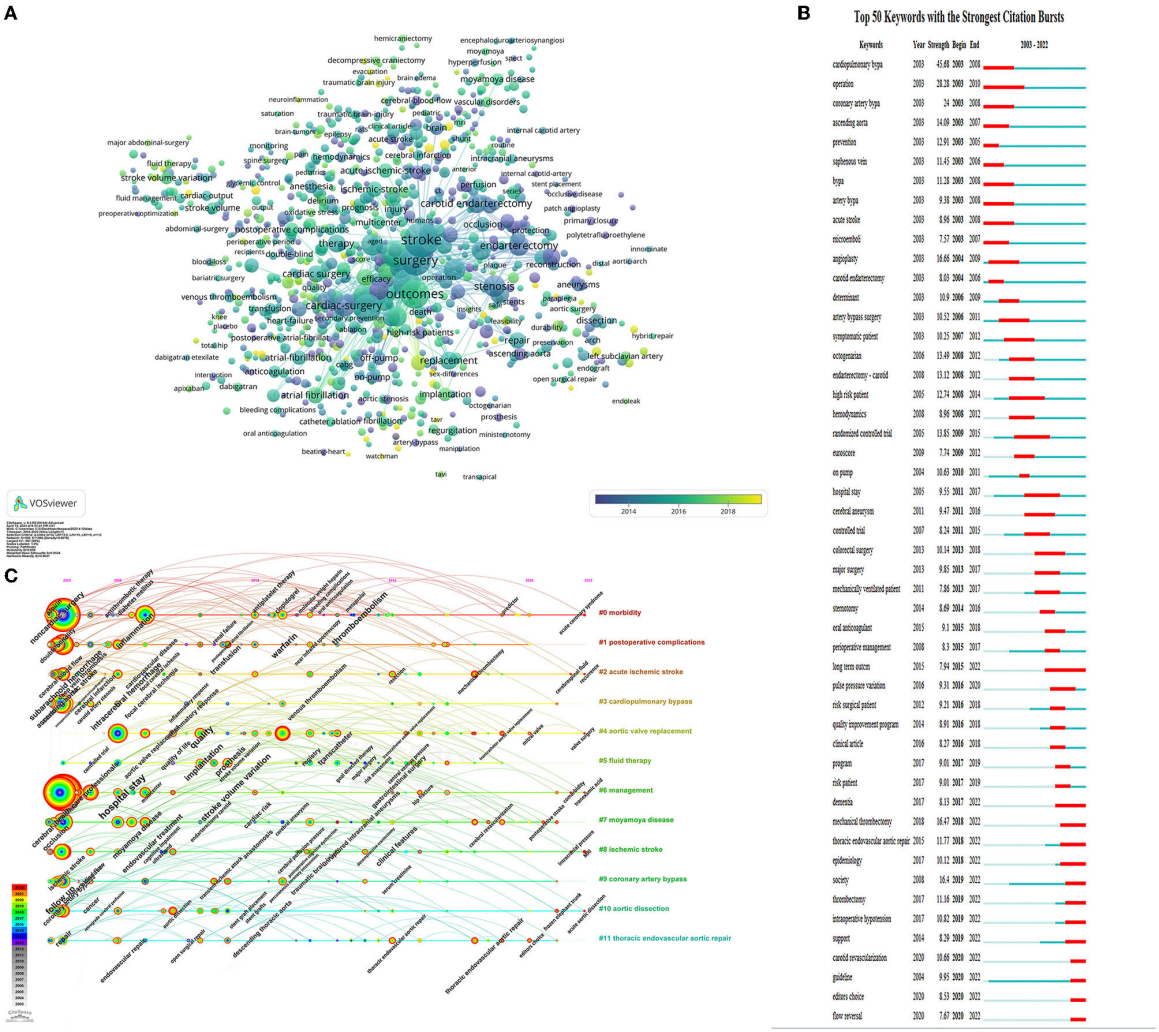
Keywords analysis **(A)** Overlay visualization of the keywords co-occurrence. The circle size indicates occurrence frequency, and a line between the circles indicates their co-occurrence in the same publication. **(B)** Top 50 keywords with the strongest citation bursts on perioperative stroke research between 2003 and 2022. The red segment of the blue line denotes the burst duration of a keyword. **(C)** CiteSpace visualization map of timeline viewer. Each node represents a keyword, and the annual ring of the node indicates the appearance time of keyword.

A list of the 20 most common keywords mentioned by perioperative stroke publications is presented in Table 5. The top five co-occurring keywords were stroke (*n* = 2,825), surgery (*n* = 1,853), outcome (*n* = 1,636), mortality (*n* = 1,436), and risk (*n* = 1,375). To visualize the global trends in perioperative stroke research over the last two decades, a visual network map of co-occurrence analysis was created. An overlay visualization map (Figure 5A) was colored using the VOSviewer according to the mean year in which the keywords appeared in a publication. The overlay visualization of the keywords, which appeared in the most recent years, included “TAVI,” “mean arterial-pressure,” “TCAR,” “predictive factors,” “thrombectomy,” “intraoperative hypotension,” “biomarker,” and “transcarotid artery revascular.”

**Table 5:** Top 20 keywords.

**Keywords**	**Number of occurrences**
Stroke	2,825
Surgery	1,853
Outcomes	1,636
Mortality	1,436
Risk	1,375
Management	1,181
Cardiac-surgery	769
Complications	756
Disease	740
Stenosis	733
Risk-factors	711
Endarterectomy	606
Impact	571
Carotid endarterectomy	511
Revascularization	506
Cardiopulmonary bypass	506
Experience	503
Meta-analysis	471
Trial	463
Therapy	425

Burst keywords were performed to predict cutting-edge research. The green line represented the timeline, and the red line represented the outbreak period. As presented in Figure 5B, the keywords with the strongest burst strength were cardiopulmonary bypass (45.68), operation (28.28), angioplasty (16.66), and mechanical thrombectomy (16.47). The burst keywords that continued until 2022 included “long-term outcome,” “thrombectomy,” “intraoperative hypotension,” “epidemiology,” “dementia,” “carotid revascularization,” “guideline,” and “flow reversal.”

A timeline view of the keywords' co-occurrence network analysis was performed with CiteSpace. The most frequent keywords for each cluster over time were visualized (Figure 5C). Each horizontal line represented a keyword cluster, which was sorted by the total number of keywords in descending order. The timeline from left to right represents 2003 to 2022. The corresponding year in which the node appears in the horizontal line represents its first appearance. The red circle refers to the keywords which appeared more recently, and the gray and blue ones represented keywords appearing in the early period.

Clusters #0 (morbidity) and #1(postoperative complications) are the two largest clusters. The keywords of “antithrombotic therapy,” “thromboembolism,” “oral anticoagulation,” “molecular weight heparin,” “metoprolol,” “renal failure,” and “diabetes mellitus” appeared only in the early period. In contrast, the keywords of “aspirin,” “antiplatelet therapy,” “clopidogrel,” “warfarin,” “bleeding complications,” “near infrared spectroscopy,” “postoperative atrial fibrillation,” and “predictor” appeared throughout the entire period. “Acute coronary syndrome” was the new emerging keyword. This result indicates that antiplatelet and antithrombosis have been hot research topics in the past 20 years. Monitoring technique with near-infrared spectroscopy has received increasing attention.

Clusters #2 (acute ischemic stroke) and #8 (ischemic stroke) refer to a similar theme. The keywords of “cerebral perfusion pressure,” “ultrasound,” “magnetic resonance,” “cognitive impairment,” and “focal cerebral ischemia” appeared only in the early period. Red nodes represented the keywords of “recurrence,” “mechanical thrombectomy,” “resection,” “MRI,” “intracranial pressure,” “postoperative cognitive dysfunction,” and “decompressive craniectomy.” The distribution of keywords indicates the shift of research focus, with mechanical thrombectomy being established as a popular treatment more recently. The association between postoperative stroke and postoperative cognitive dysfunction has always been the research hotspot in this field.

Clusters #3 (cardiopulmonary bypass), #4 (aortic valve replacement), #9 (coronary artery bypass), and #11 (thoracic endovascular aortic repair) are related to cardiovascular surgery. The keywords of “intraoperative transesophageal echocardiography”, “open surgical repair”, “stent grafts”, “inflammatory response”, and “serum creatinine” appeared in the early period. More recent studies have focused on minimally invasive and interventional cardiac procedures as evidenced by the emerging keywords of “carotid artery stenosis,” “transcatheter aortic valve replacement,” “transient ischemic attack,” “percutaneous coronary intervention,” and “thoracic endovascular aortic repair.”

In clusters #5 (fluid therapy) and #6 (management), the keywords included “risk assessment,” “goal directed therapy,” “controlled trial,” and “major surgery” appeared in the earlier period. New emerging keywords included “central venous pressure,” “stroke volume variation,” “multicenter,” “hip fracture,” and “tranexamic acid.” Hip fracture and tranexamic acid have been shown to be associated with an increased risk of postoperative stroke in surgical patients, these terms might represent current hot research topics.

In cluster #7 (moyamoya disease), early keywords included “cerebral aneurysms,” and “endarterectomy carotid.” The keywords “postoperative stroke” and “cerebral revascularization” appeared in more recent periods. In cluster #10 (aortic dissection), “frozen elephant trunk” is the new emerging keyword.

## 4. Discussion

### 4.1. Global trends

The bibliometric properties of 10,172 documents—which were retrieved from the WoS database by this study—in the citation indices were scrutinized. Over the past two decades, the number of perioperative stroke papers had almost tripled from 226 to 905. After 2018, a sharp increase was observed, and the number is projected to increase to 1,267 by 2032, indicating that the condition was a popular research topic. An increase in the number of publications could be associated with the growing medical and economic burden attributed to perioperative neurological complications, which predicts a continuation of comprehensive and in-depth research into perioperative stroke.

### 4.2. Countries

The H-index and the total number of citations are essential metrics to measure the academic impact and quality of publications (11). According to the network visualization map, the 10 countries with the highest number of publications included five European countries (Germany, Italy, England, Netherlands, and France), three Asian countries (China, Japan, and South Korea), and two North American countries (the USA and Canada). The USA led the field with the highest number of citations (*n* = 114,598) and H-index (132). Although Canada contributed only 5.9% to the total number of publications, it had the highest mean number of citations per term with an outstanding H-index of 84.

According to the publication trend in every country (Figure 2B) and the overlay visualization of co-authorship analysis of countries (Figure 3B), China had the fastest-growing number of publications and had strengthened its academic collaboration with other countries. This may be attributed to Chinese extensive population base, the progress of population aging, and increasing funding in scientific research, which drive in-depth research in this field. However, its relatively low H-index (48) and mean citation figure (*n* = 10.77) indicate that its research quality can be further improved. African and Southeast Asian countries contributed minimal research to the field, which may be attributed to their lower economic growth and international academic collaborations.

### 4.3. Institutions and authors

Harvard University, the University of California System, and the Mayo Clinic were the three most prolific institutions. Eight of the 10 most prolific institutions were from the USA. Based on the co-authorship analysis, we observed that institutions from the USA were at the center of the collaborative network. Institutions in North America and Europe had close collaborations; however, there was only internal collaboration in most institutions in Asian countries (Figure 4A).

Malas, Mahmoud B., Biancari, Fausto, Schermerhorn, Marc L., and De Borst, Gert J. were the most prolific contributors to perioperative stroke research. Eight of the 10 most prolific authors were from the USA. According to the co-authorship analysis, authors from the same country had frequent close collaborations; however, the connection among authors from different countries remained low (Figure 4B). Therefore, it is recommended that scholars in different countries should overcome academic barriers and enhance cooperation to promote the development of perioperative research.

### 4.4. Journals

Journal and co-citation analysis provide important insights for researchers to guide their selection of appropriate journals for publication consideration. The co-citation network demonstrated that Stroke (IF = 10.17, 2021) had the highest recognition and authority in perioperative stroke research. It was followed by the Annals of Thoracic Surgery (IF = 5.11, 2021) and the Journal of Vascular Surgery (IF = 4.86, 2021), both of which had the highest number of publications and a fairly high TLS in the co-citation visualization network.

Based on the co-citation frequency, these journals were classified into four clusters with similar research directions (Figure 4C). The yellow cluster represented journals that dealt with vascular surgery; the blue cluster represented journals that focused on cardiac and thoracic surgery; the red cluster referred to journals that featured anesthesia and critical care; and the green cluster represented journals that focused on neurosurgery and stroke. In addition, the proportion of publications in the 10 most prolific journals was as high as 29%, indicating a dense distribution of publications across journals. This finding may be attributed to a spike in surgical, vascular, thoracic, and stroke research. Among the 10 journals, only two had an impact factor above five. These findings suggest that most studies were published by specialist journals, and greater recognition by high-impact medical journals is required.

### 4.5. Current knowledge and trending topics

#### 4.5.1. Preoperative risks and prevention strategies

Several studies have identified advanced age, renal disease, and prior episodes of transient ischemic attack or stroke as the main risk factors in perioperative stroke (3, 12, 13). Other risk factors were established by recent studies and include myocardial infarction in the last 6 months, previous history of stroke, atrial fibrillation, hypertension, chronic obstructive pulmonary disease, smoking habit, patent foramen ovale (14), female sex, and diabetes (3, 12, 15).

In addition to the identification and elucidation of the risk factors, prediction models have been used to assess perioperative stroke risk. A recent observational study found that American College of Surgeons (Chicago, Illinois) surgical risk calculator and Myocardial Infarction or Cardiac Arrest risk score, showed the highest discriminatory ability for predicting a likelihood of perioperative strokes (16). However, most prediction tools failed to account for stroke risk associated with specific scores or thresholds. Consequently, a prediction model must be developed, which comprehensively integrates every relevant risk factor and provides quantitative data for stroke risk stratification.

Preoperative medication optimization is essential for reducing adverse perioperative outcomes. Although some studies have demonstrated that beta-blockers—such as metoprolol—do not reduce the risk of perioperative stroke (17, 18), there does not appear to be an association between chronic beta-blockers and perioperative stroke in patients with multiple cardiovascular risk factors, such as hypertension (19), previous coronary revascularization (20), and diabetes mellitus (21). For patients on statins before undergoing non-cardiac surgery, it is recommended that they continue to use the drugs to reduce adverse cardiovascular events; however, the relationship between statins and stroke risk is not clearly defined (22, 23).

Anti-coagulation strategies for surgical patients should balance the risk of thromboembolic prevention and the increased risk of surgical bleeding. Perioperative anticoagulation bridging therapy is only indicated in high-risk thromboembolic patients who are on vitamin K antagonists, such as warfarin (24). For oral anticoagulants, it is recommended to discontinue these drugs 2–3 days before surgery and resume them 1–3 days post-operation, based on the risk of thromboembolism and bleeding. In patients who have not undergone percutaneous coronary intervention, the use of low-dose aspirin must be discontinued as it can lead to an increased risk of perioperative bleeding (25).

In addition, preoperative functional magnetic resonance imaging can be performed in high-risk patients with previous cerebrovascular disease to determine cerebrovascular reserve (26). This preoperative strategy helps identify individualized intraoperative management goals for blood pressure and end-tidal carbon dioxide to optimize cerebrovascular perfusion.

#### 4.5.2. Intraoperative management

Current research suggests that general or regional anesthesia does not affect perioperative stroke risk (27). The choice of anesthesia must be based on other clinical indications rather than the perioperative stroke risk. With the possible exception of patients undergoing hip surgery, neuraxial anesthesia appears to reduce stroke risk compared to general anesthesia (28).

Although the literature findings are inconclusive, arterial blood pressure may play an important role in perioperative stroke (29). Nevertheless, the lack of a well-defined threshold for intraoperative blood pressure indicates an increased risk of perioperative stroke in patients undergoing non-cardiac surgery. Until a specific threshold is clarified, mean arterial pressure should be maintained above 70 mmHg to reduce the risk of perioperative stroke, particularly in high-risk patients (30). Intraoperative strategies based on near-infrared spectroscopy and bioimpedance can be used to determine critical thresholds for cerebral blood flow (31, 32).

Although the FEDORA trial (clinical trial registration: ISRCTN93543537) found a reduction of almost 50% in moderate or severe postoperative complications in patients who were randomly assigned to intraoperative goal-directed therapy, it did not describe a significant reduction in stroke, which was attributed to its low incidence (33). In addition, both hypoventilation and hyperventilation may lead to a reduction in cerebral blood flow that reaches the threshold of hypoxic-ischemic injury (34). An optimization of ventilation strategies is another means to improve outcomes. Despite the absence of data that support goal-directed therapy and lung-protective ventilation in stroke prevention, these methods seem reasonable as part of an overall strategy to improve perioperative outcomes.

#### 4.5.3. Postoperative recognition and stroke treatment

In the perioperative period, stroke diagnosis is challenging owing to the use of opioids and sedatives, pain, and delayed neurocognitive recovery. Although various clinical screening tools are available to detect stroke (35), the postoperative scores obtained by these scales—such as the modified National Institutes of Health (Bethesda, MD, USA) stroke scale—are usually altered compared with the baseline (36). In the absence of reliable clinical screening tools, serum biomarkers can be used to assist in the assessment and diagnosis of perioperative stroke. However, to date, no reliable biomarkers have been clinically validated for the identification of cerebral ischemia and infarction (36). Based on postoperative signs and symptoms of large-vessel occlusion, targeted monitoring that uses computed tomography or magnetic resonance imaging can improve stroke diagnosis.

When a perioperative stroke is suspected, non-contrast computed tomography or magnetic resonance imaging must be performed to determine whether its etiology is hemorrhagic or ischemic (2). When these criteria are met, the recommended guidelines of intravenous alteplase for thrombolytic therapy must be instituted within 4.5 h from symptom onset. Thereafter, dosing may be used in certain patients (37). However, owing to the potential adverse effects of thrombolytic therapy, the time window and treatment indications must be strictly controlled. As mechanical thrombectomy is superior to intravenous thrombolysis (38), its use as a treatment option must be prioritized. Current guidelines support its intervention in patients experiencing symptom onset within 6–24 h (37).

#### 4.5.4. Future directions and research gap

Despite steady progress that has been achieved in the research of perioperative stroke in the past two decades, the research gaps remain to be explored in this specific field. The pathophysiology of perioperative stroke needs to be further clarified elaborately, which appears to be distinct from a non-surgical stroke. In addition, balancing the risk of embolism formation and postoperative bleeding in surgical patients can be challenging. Refinement of the patients at high risk of perioperative embolization or bleeding was required before the prescription of bridging treatment. Furthermore, the biomarker-based diagnosis of a perioperative stroke may greatly improve diagnostic efficiency; however, no reliable biomarkers are currently available. More specific and refined prediction models including the relevant risk factors should be developed in predicting the likelihood of perioperative stroke. As intraoperative blood pressure reduction and hyperventilation can decrease the cerebral blood flow to the hypoxic threshold, an analysis of the intraoperative physiological data, provided by a multicenter database, can be used to determine an association between hypotension and end-tidal carbon dioxide with stroke. In addition, the role of intraoperative management strategies—such as the optimization of blood pressure and glucose—in stroke patients deserves further study. Finally, the potential outcomes of perioperative stroke on postoperative delirium and cognitive dysfunction and the association between ischemic lesions with the perioperative neurocognitive disorder are yet to be clarified.

### 4.6. Limitations

This study has several limitations. First, the bibliometric analysis was based on the data retrieved from the WoS database, and only English publications were included. Both factors led to selection bias, and several important publications may have been omitted in the process. Second, owing to a lack of author and address details in the publications, a count of the author collaborations may lack accuracy. Finally, although the number of citations is often used as an indicator of publication quality, this index is likely to be affected by self-citation and publication date. As recent publications tend to have a lower number of citations, their impact may have been underestimated.

### 4.7. Conclusion

In this study, a summary and a visual map of perioperative stroke research over the past two decades were provided to illustrate the current status, hotspots, and development of this field. Research on perioperative antiplatelet and antithrombotic, cardiovascular surgery, postoperative cognitive dysfunction, thrombectomy, tranexamic acid, and frozen elephant trunk has attracted increasing attention, and these topics are emerging hotspots of present research and possible candidates for future research.

## Data availability statement

The original contributions presented in the study are included in the article/supplementary material, further inquiries can be directed to the corresponding authors.

## Author contributions

BY and TT: conception and design of the study. SJ: literature search and paper writing. YS: VOSviewer and CiteSpace analysis. XF and TJ: figure and table drawing. XY: manuscript review and editing. All authors contributed to the manuscript and approved the submitted version.

## References

[1] Sacco RL Kasner SE Broderick JP Caplan LR Connors JJ Culebras A . An updated definition of stroke for the 21st century: a statement for healthcare professionals from the american heart association/american stroke association. Stroke. (2013) 44:2064–89. 10.1161/STR.0b013e318296aeca 23652265 PMC11078537

[2] Vlisides PE Moore LE Whalin MK Robicsek SA Gelb AW Lele AV . Perioperative care of patients at high risk for stroke during or after non-cardiac, non-neurological surgery: 2020 guidelines from the society for neuroscience in anesthesiology and critical care. J Neurosurg Anesthesiol. (2020) 32:210–26. 10.1097/ANA.0000000000000686 32433102

[3] Mashour GA SA Kheterpal S. Perioperative stroke and associated mortality after noncardiac, nonneurologic surgery. Anesthesiology. (2011) 114:1289–96. 10.1097/ALN.0b013e318216e7f4 21478735

[4] Neuro VI. Perioperative covert stroke in patients undergoing non-cardiac surgery (neurovision): a prospective cohort study. Lancet. (2019) 394:1022–9. 10.1016/S0140-6736(19)31795-7 31422895

[5] Smilowitz NR Gupta N Ramakrishna H Guo Y Berger JS Bangalore S. Perioperative major adverse cardiovascular and cerebrovascular events associated with noncardiac surgery. JAMA Cardiol. (2017) 2:181–7. 10.1001/jamacardio.2016.4792 28030663 PMC5563847

[6] Tan HH Xu J Teoh HL Chan BP Seet RC Venketasubramanian N . Decline in changing montreal cognitive assessment (moca) scores is associated with post-stroke cognitive decline determined by a formal neuropsychological evaluation. PLoS ONE. (2017) 12:e0173291. 10.1371/journal.pone.0173291 28346532 PMC5367691

[7] Saltman AP Silver FL Fang J Stamplecoski M Kapral MK. Care and Outcomes of Patients with in-Hospital Stroke. JAMA Neurol. (2015) 72:749–55. 10.1001/jamaneurol.2015.0284 25938195

[8] Wang K Xing D Dong S Lin J. The global state of research in nonsurgical treatment of knee osteoarthritis: a bibliometric and visualized study. BMC Musculoskelet Disord. (2019) 20:407. 10.1186/s12891-019-2804-9 31484517 PMC6727547

[9] Kumar S. Co-Authorship networks: a review of the literature. Aslib J. Inf. Manage. (2015) 67:55–73. 10.1108/AJIM-09-2014-0116

[10] Kleminski R Kazienko P Kajdanowicz T. Analysis of direct citation, co-citation and bibliographic coupling in scientific topic identification. J. Inf. Sc. (2020) 48:349–73. 10.1177/016555152096277536833921

[11] Bastian S Ippolito JA Lopez SA Eloy JA Beebe KS. The use of the H-index in academic orthopaedic surgery. J Bone Joint Surg Am. (2017) 99:e14. 10.2106/JBJS.15.01354 28196042

[12] Sutzko DC Andraska EA Obi AT Henke PK Osborne NH. Risk factors associated with perioperative myocardial infarction in major open vascular surgery. Ann Vasc Surg. (2018) 47:24–30. 10.1016/j.avsg.2017.08.030 28893702 PMC5805566

[13] Sharifpour M Moore LE Shanks AM Didier TJ Kheterpal S Mashour GA. Incidence, predictors, and outcomes of perioperative stroke in noncarotid major vascular surgery. Anesthesia Analgesia. (2013) 116:424–34. 10.1213/ANE.0b013e31826a1a32 23115255

[14] Friedrich S Ng PY Platzbecker K Burns SM Banner-Goodspeed V Weimar C . Patent foramen ovale and long-term risk of ischaemic stroke after surgery. Eur Heart J. (2019) 40:914–24. 10.1093/eurheartj/ehy402 30020431 PMC6416532

[15] Vasivej T Sathirapanya P Kongkamol C. Incidence and risk factors of perioperative stroke in noncardiac, and nonaortic and its major branches surgery. J Stroke Cerebrovasc Dis. (2016) 25:1172–6. 10.1016/j.jstrokecerebrovasdis.2016.01.051 26922129

[16] Wilcox T Smilowitz NR Xia Y Berger JS. Cardiovascular risk scores to predict perioperative stroke in noncardiac surgery. Stroke. (2019) 50:2002–6. 10.1161/STROKEAHA.119.024995 31234757 PMC10027603

[17] Group PS Devereaux PJ Yang H Yusuf S Guyatt G Leslie K . Effects of extended-release metoprolol succinate in patients undergoing non-cardiac surgery (poise trial): a randomised controlled trial. Lancet. (2008) 371:1839–47. 10.1016/S0140-6736(08)60601-7 18479744

[18] Hajibandeh S Hajibandeh S Antoniou SA Torella F Antoniou GA. Effect of beta-blockers on perioperative outcomes in vascular and endovascular surgery: a systematic review and meta-analysis. Br J Anaesth. (2017) 118:11–21. 10.1093/bja/aew380 28039238

[19] Jørgensen ME Hlatky MA Køber L Sanders RD Torp-Pedersen C Gislason GH . β-Blocker–associated risks in patients with uncomplicated hypertension undergoing noncardiac surgery. JAMA Inte. Med. (2015) 175:5346. 10.1001/jamainternmed.2015.5346 26436291

[20] Lopez-Delgado JC Park J Kim J Kwon JH Park SJ Min JJ . Association between perioperative β-blocker use and clinical outcome of non-cardiac surgery in coronary revascularized patients without severe ventricular dysfunction or heart failure. Plos ONE. (2018) 13:311. 10.1371/journal.pone.0201311 30067841 PMC6070245

[21] Chen RJ Chu H Tsai LW. Impact of beta-blocker initiation timing on mortality risk in patients with diabetes mellitus undergoing noncardiac surgery: a nationwide population-based cohort study. J Am Heart Assoc. (2017) 6:4392. 10.1161/JAHA.116.004392 28073770 PMC5523631

[22] Ma B Sun J Diao S Zheng B Li H. Effects of perioperative statins on patient outcomes after noncardiac surgery: a meta-analysis. Ann Med. (2018) 50:402–9. 10.1080/07853890.2018.1471217 29741972

[23] London MJ Schwartz GG Hur K Henderson WG. Association of perioperative statin use with mortality and morbidity after major noncardiac surgery. JAMA Intern Med. (2017) 177:231–42. 10.1001/jamainternmed.2016.8005 27992624

[24] Douketis JD Spyropoulos AC Kaatz S Becker RC Caprini JA Dunn AS . Perioperative bridging anticoagulation in patients with atrial fibrillation. New England J. Med. (2015) 373:823–33. 10.1056/NEJMoa1501035 26095867 PMC4931686

[25] Devereaux PJ Mrkobrada M Sessler DI Leslie K Alonso-Coello P Kurz A . Aspirin in patients undergoing noncardiac surgery. New England J. Med. (2014) 370:1494–503. 10.1056/NEJMoa1401105 24679062

[26] Vlisides PE Moore LE. Stroke in surgical patients. Anesthesiology. (2021) 134:480–92. 10.1097/ALN.0000000000003664 33411913

[27] Christiansen MN. Risks of cardiovascular adverse events and death in patients with previous stroke undergoing emergency noncardiac, nonintracranial surgery: the importance of operative timing. Anesthesiology. (2017) 127:9–19. 10.1097/ALN.0000000000001685 28514242

[28] Chu CC. Propensity score-matched comparison of postoperative adverse outcomes between geriatric patients given a general or a neuraxial anesthetic for hip surgery: a population-based study. Anesthesiology. (2015) 123:136–47. 10.1097/ALN.0000000000000695 25955981

[29] Hsieh JK Dalton JE Yang D Farag ES Sessler DI Kurz AM. The association between mild intraoperative hypotension and stroke in general surgery patients. Anesth Analg. (2016) 123:933–9. 10.1213/ANE.0000000000001526 27636576

[30] Benesch C Glance LG Derdeyn CP Fleisher LA Holloway RG Messe SR . Perioperative neurological evaluation and management to lower the risk of acute stroke in patients undergoing noncardiac, nonneurological surgery: a scientific statement from the american heart association/american stroke association. Circulation. (2021) 143:e923–e46. 10.1161/CIR.0000000000000968 33827230

[31] Joshi B Ono M Brown C Brady K Easley RB Yenokyan G . Predicting the limits of cerebral autoregulation during cardiopulmonary bypass. Anesth Analg. (2012) 114:503–10. 10.1213/ANE.0b013e31823d292a 22104067 PMC3288415

[32] Tiba MH MB Ansari S Belle A Cummings BC Rajajee V Patil PG . Novel noninvasive method of cerebrovascular blood volume assessment using brain bioimpedance. J Neurotrauma. (2017) 34:3089–96. 10.1089/neu.2017.5090 28657491

[33] Calvo-Vecino JM Ripolles-Melchor J Mythen MG Casans-Frances R Balik A Artacho JP . Effect of goal-directed haemodynamic therapy on postoperative complications in low-moderate risk surgical patients: a multicentre randomised controlled trial (fedora trial). Br J Anaesth. (2018) 120:734–44. 10.1016/j.bja.2017.12.018 29576114

[34] Fisher JA VL Mikulis DJ. Magnetic resonance imaging-based cerebrovascular reactivity and hemodynamic reserve. Stroke. (2018) 49:2011–8. 10.1161/STROKEAHA.118.021012 29986929

[35] Sun Z YY Leung CC Chan MT Gelb AW. Study group for perioperative stroke in china (posic). clinical diagnostic tools for screening of perioperative stroke in general surgery: a systematic review. Br J Anaesth. (2016) 116:328–38. 10.1093/bja/aev452 26821695

[36] Vlisides PE Kunkler B Thompson A Zierau M Lobo R Strasser MO . Cerebrovascular disease and perioperative neurologic vulnerability: a prospective cohort study. Front Neurol. (2019) 10:560. 10.3389/fneur.2019.00560 31231299 PMC6558425

[37] Powers WJ Rabinstein AA Ackerson T Adeoye OM Bambakidis NC Becker K . Guidelines for the early management of patients with acute ischemic stroke: 2019 update to the 2018 guidelines for the early management of acute ischemic stroke: a guideline for healthcare professionals from the american heart association/american stroke association. Stroke. (2019) 50:e344–418. 10.1161/STR.0000000000000211 31662037

[38] Goyal M Menon BK van Zwam WH Dippel DW Mitchell PJ Demchuk AM . Endovascular thrombectomy after large-vessel ischaemic stroke: a meta-analysis of individual patient data from five randomised trials. Lancet. (2016) 387:1723–31. 10.1016/S0140-6736(16)00163-X 26898852

